# Application of Porcine Kidney-Derived Extracellular Matrix as Coating, Hydrogel, and Scaffold Material for Renal Proximal Tubular Epithelial Cell

**DOI:** 10.1155/2022/2220641

**Published:** 2022-01-28

**Authors:** Eun Hye Lee, So Young Chun, Bo Hyun Yoon, Hyun Tae Kim, Jae-Wook Chung, Jun Nyung Lee, Yun-Sok Ha, Tae Gyun Kwon, Kyeong-Hyeon Byeon, Bum Soo Kim

**Affiliations:** ^1^Joint Institute for Regenerative Medicine, Kyungpook National University, Daegu, Republic of Korea; ^2^BioMedical Research Institute, Kyungpook National University Hospital, Daegu, Republic of Korea; ^3^Department of Urology, School of Medicine, Kyungpook National University, Kyungpook National University Hospital, Daegu, Republic of Korea; ^4^Department of Urology, School of Medicine, Kyungpook National University, Kyungpook National University Chilgok Hospital, Daegu, Republic of Korea; ^5^Department of Urology, Haewoo Urology Clinic, Daegu, Republic of Korea

## Abstract

**Background:**

Human renal proximal tubular epithelial (RPTE) cell is a very useful tool for kidney-related experiments in vitro/ex vivo. However, only a few primary RPTE cells can be obtained through kidney biopsy, the proliferation rate of primary cell is very low, and the cultured cell properties are easily altered in artificial conditions. Thus, RPTE cell usage is very tricky; we applied porcine kidney-derived extracellular matrix (renal ECM) as coating, hydrogel, and scaffold material to increase cell proliferation and maintain cellular properties providing three-dimensional (3D) niche, which can be a valuable cell delivery vehicle.

**Methods:**

Porcine renal ECM was prepared by decellularization using 1% Triton X-100, solubilized with 0.5 M acetic acid. The final protein concentration was adjusted to 10 *μ*g/*μ*L (pH 7.0). The efficacies as coating, hydrogel, and scaffold materials were analyzed through cell morphology, proliferation rate, renal-associated gene expressions, chemical composition, and microstructure evaluation. The efficacies as a coating material were compared with Matrigel, collagen type 1 (col1), gelatin, fibrinogen, and thrombin. After confirmation of coating effects, the effective concentration range was decided. The efficacies as hydrogel and scaffold materials were compared with hyaluronic acid (HA) and col1, respectively.

**Results:**

As the coating material, renal ECM showed a higher cell proliferation rate compared to other materials, except for Matrigel. Renal-associated gene expressions were significantly enhanced in the renal ECM than other materials. Coating effect on cell proliferation was dependent on the renal ECM concentration, and the effective concentration ranged from 30 to 100 *μ*g. As the hydrogel material, renal ECM showed a distinct inner cell network morphology and significantly increased renal-associated gene expressions, compared to HA hydrogel. As the scaffold material, renal ECM showed specific amide peaks, enhanced internal porosity, cell proliferation rate, and renal-associated gene expression compared to the col1 scaffold.

**Conclusions:**

We concluded that renal ECM can be a suitable material for RPTE cell culture and usage. More practically, the coated renal ECM stimulates RPTE cell proliferation, and the hydrogel and scaffold of renal ECM provide useful 3D culture niche and cell delivery vehicles maintaining renal cell properties.

## 1. Introduction

Acute or chronic kidney disease has become one of the major global health problems, due to aging and comorbid diseases such as diabetes and hypertension. Kidney disease is caused by loss of renal parenchyma, fibrosis, capillary rarefaction, and blood filtering disfunction [1]. To treat kidney disease, cell-based therapy, pathophysiological in vivo-mimic models, and three-dimensional (3D) kidney structure simulations are being investigated. For success of these experiments, the most important factor is the cell that is necessary to secure a sufficient cell number and maintain the unique cell properties.

Recently, adult mammalian kidneys get attention for their regenerative ability through renal progenitor cell existence [2], mature tubular epithelium's dedifferentiation, and epithelial-to-mesenchymal transition (EMT) phenomenon [3, 4]. These new findings in the adult kidney frequently come from the renal proximal tubule [5]. The cells isolated from the renal proximal tubule have stemness and can differentiate into several types of renal lineage cells, which is an important property considering the kidney is a complex organ with various cell types. Thus, the renal proximal tubule epithelial (RPTE) cell is one of the major autologous cell sources for kidney experiments in vitro/ex vivo and in vivo therapies [6].

However, it is not easy to get enough cell number from a small biopsy, and the proliferation rate of RPTE cell is very low because they pause in the G1 cell cycle [7]. Also, the primary cultured cell populations are very heterogeneous, with many fully differentiated epithelial cells and a small portion of progenitors. During in vitro/ex vivo culture, mature RPTE cells' intrinsic properties (such as fluid blockade, selective absorption, secretion, ion transport, and transcytosis) [8–10] are gradually altered with passages. Besides, progenitor cells may differentiate into spontaneous directions. For these reasons, the cell quality assurance is not easy; even commercial RPTE cells warrant less than 15 passages (#ATCC, PCS-400-010). To overcome these problems, we propose the use of porcine renal-derived decellularized ECM. As already reported by us [11], this material contains kidney-specific ECM (laminin, collagen, and fibronectin) and bioactive molecules (VEGF, IGF-1, and HGF), which are essential components for renal cytology and functionality. We expect the renal ECM can provide a biomimetic niche for RPTE cells in vitro/ex vivo culture and in vivo delivery.

To provide an optimized biomimetic niche for RPTE cell, we prepared renal ECM as three different phases: coating, hydrogel, and scaffold. According to the ECM phase (dimension, stiffness, porosity, etc.), cell responses can be changed. For example, depending on the attachment surface, cell shape is changed, and by the cell shape, the cell membrane receptors are exposed to different patterns. The exposed receptors showed a diverse activity, which results to different transcriptional sequences, and finally, cell function is altered [12]. To verify renal ECM efficiency as a coating material, ECM was coated to the culture dish, and cell responses were compared with commercial products, such as Matrigel, collagen type 1 (col1), gelatin, fibrinogen, and thrombin. To verify renal ECM efficiency as 3D cultures and delivery devices, hydrogelated and lyophilized renal ECM were manufactured, and cell responses were compared to the hyaluronic acid (HA) hydrogel and collagen scaffold, respectively. Therefore, by evaluating of RPTE cell responses, we estimate the effectiveness of renal ECM as a coating, hydrogel, and scaffold materials for cell proliferation and property maintenance, providing 3D niche and cell delivery vehicle.

## 2. Materials and Methods

### 2.1. Porcine Renal ECM Preparation

Pig kidneys (2–3 months, 22–30 kg) were excised, and the cortex area was separated. The cortex tissue was washed with PBS twice and treated with 1% Triton X-100 (Sigma-Aldrich, St. Louis, MO, USA) for decellularization and centrifuged at 150 rpm at 4°C. The decellularization solution was changed every 4 hrs on the first day and every 24 hrs after that for 1–1.5 weeks. When the tissue became transparent, complete decellularization was confirmed with Hematoxylin and Eosin and DAPI (Santa Cruz Biotechnology, Santa Cruz, CA, USA) staining. After complete washing, the decellularized renal ECM was freeze-dried and sterilized with ethylene oxide gas. For renal ECM solubilization, ECM was soaked in 0.5 M acetic acid and placed in a heating block at 80°C for 5 min. After confirmation of melting, the total protein concentration was adjusted to 10 *μ*g/*μ*L and pH 7.0.

### 2.2. Cell Preparation

For cell quality guarantee, the commercial RPTE cell was purchased from ScienCell Research Laboratories (Carlsbad, CA, USA). The cells were cultured on a tissue culture plate (Costar, Northwest Washington, DC, USA) in a basal epithelial cell medium with 1% epithelial cell growth supplement (ScienCell Research Laboratories) at 37°C in a 5% CO_2_ atmosphere. Cells were subcultured about 80% confluence and used for the experiments at passage 10.

### 2.3. Analysis of Renal ECM as a Coating Material

To verify renal ECM efficiency as a coating material, it was compared with commercially available coating materials, Matrigel (10 mg/mL, #356255, Corning), rat tail collagen type 1 (col1., 3–4 mg/mL, #354236, Corning), gelatin (1 mg/mL, Cellrix#B1010-024, Medifab, Rolleston, New Zealand), fibrinogen (95 mg/mL, Greenplast, Greencross, Seoul, Korea), and thrombin (0.53 mg/mL, Greenplast, Greencross). 100 *μ*g of each material was placed in each well of a 48-well plate (clear, flat-bottom, Corning) and kept at 4°C for 12 hrs. The negative control was an uncoated polystyrene surface. Each condition was prepared in triplicate. Cells were seeded with 1 × 10^3^ per well of a 48-well plate. For 7 days, cells were observed under a light microscope for cell morphological appearance and extracted at each time point for proliferation and gene expression analysis.

For analysis of the renal ECM concentration effect for RPTE cell proliferation, renal ECM (10, 30, 50, or 100 *μ*g) was added to each well of a 48-well plate and kept at 4°C for 12 hrs. Each condition was prepared in triplicate. Cells were seeded with 1 × 10^3^ per well. The cells were examined under a light microscope for morphological appearance for 7 days and extracted cells for analysis of the proliferation rate and gene expression at each time point.

### 2.4. Analysis of Renal ECM as a Hydrogel Material

To prepare the hydrogel phase, 1 mg of renal ECM was added to each well of an 8-well chamber slide (MatTek, Bratislava, Slovak), and 1 × 10^3^ cells were added to each well. After gently mixing with a pipette, the medium was added and cultured for 7 days. HA hydrogel (50 mg/mL, HyStem#GS1004, Advanced BioMatrix, Carlsbad, CA, USA) was used as control. The HA hydrogel was prepared according to the manufacturer's protocol. The HA concentration and cell seeding procedure were the same as with the renal ECM hydrogel. Each condition was prepared in triplicate.

### 2.5. Analysis of Renal ECM as a Scaffold Material

Renal ECM solution (1 mg) was placed on the flat plate (mold size, 5 × 2.5 × 1 mm^3^), frozen for 24 hrs at −80°C, then lyophilized for 24 hrs using a freeze dryer (Bondiro, #FD8508, Ilshin, Korea). Commercial col1 (Corning) was prepared as a control, using the same procedure as used for the renal scaffold. After 24 hr sterilization with UV light, the prepared scaffolds were placed into each well of a 48-well plate and soaked in the culture medium for 5 min before adding 1 × 10^3^ cells to each well.

### 2.6. Cell Proliferation and Senescence Assay

Cell proliferation was evaluated using a 3-[4,5-dimethylthiazol-2-yl]-2,5-diphenyltetrazolium bromide (MTT) assay (Sigma-Aldrich). Each well had 0.5 mg/mL of MTT solution added and was incubated for 2 hrs at 37°C. The formazan crystals in the cells were solubilized with 0.11 mL of dimethyl sulfoxide. An aliquot of each sample (100 *μ*L) was transferred to 96-well plates, and the absorbance was measured at 550 nm using an ELISA Reader (BioTek, Winooski, VT, USA).

For the senescence assay, cells were washed with PBS, and 100 *μ*L of 1× fixative solution was added to each well, which was incubated for 10–15 min at room temperature. The fixative solution was removed, and the cells were washed 3 times with PBS. After adding 100 *μ*L staining solution to each well, the plate was incubated for several minutes in a moist chamber at 37°C, until stained blue. The staining solution was removed, and the cells were washed 3 times with PBS and examined with a light microscope. The positive cells versus the total number of cells were counted to calculate the percent of SA-*β*-gal-positive cells, and representative pictures were taken at 100x magnification.

### 2.7. Renal-Associated Gene Expression

For cell isolation from the hydrogel and scaffold, they were treated with collagenase and hyaluronidase (Sigma-Aldrich). The total RNA was extracted with a RNeasy-kit (Qiagen, Hilden, Germany) according to the manufacturer's instructions. A total of 2 g of RNA was used for cDNA synthesis using cDNA reverse transcription kits (Applied Biosystems, Warrington, UK). The primers were designed with Primer Express Software (Applied Biosystems). The assay was performed using the ABI Prism Sequence Detection System 7800 with SYBR Green Polymerase Chain Reaction Master Mix (Applied Biosystems). For analysis, the 2^−△△Ct^ method of relative quantification was adapted to estimate the copy numbers. The primer sequences are listed in Table 1. The human kidney-specific progenitors (CD133, CD24), representative renal progenitor (Pax2), and general physiological feature (pan-Cytokeratin) were surveyed. The epithelial cell properties were evaluated with endocytosis receptor (Megalin), ion channels (Muc-1), water channel (AQP1), and sodium-dependent glucose transport system (SGLT2). The dedifferentiation from final differentiated epithelium to the renal progenitor state and the EMT were evaluated with E-cadherin, *β*-catenin, and Vimentin.

### 2.8. Chemical/Physical Analysis

For chemical composition analysis of the scaffolds, Fourier transform infrared (FTIR) spectroscopy (Thermo Scientific, Waltham, MA, USA) was performed with a spectral range of 400−4000 cm^−1^ in transmittance mode. An expert operator assessed the surface property and microstructural morphology of the scaffold with field emission scanning electron microscopy (FE-SEM, S-4300, Hitachi, Tokyo, Japan). For cytophilic evaluation at each time point, the cell-scaffold complex was fixed with 10% neutral formalin, paraffin format; sliced into 5 *μ*m thicknesses; and stained with DAPI (Santa Cruz Biotechnology). The cell distribution on the scaffold was examined with a fluorescence microscope (Nikon, Tokyo, Japan).

### 2.9. Statistical Analyses

All values are expressed as means ± SD. Statistical analyses were performed with Student's *t*-test (*p* values) to compare cell proliferation and intrinsic properties among the different tested culture systems, with significance being *p* < 0.05.

## 3. Results

### 3.1. Cell Responses to the Renal ECM Coating Material

**Figure 1:**
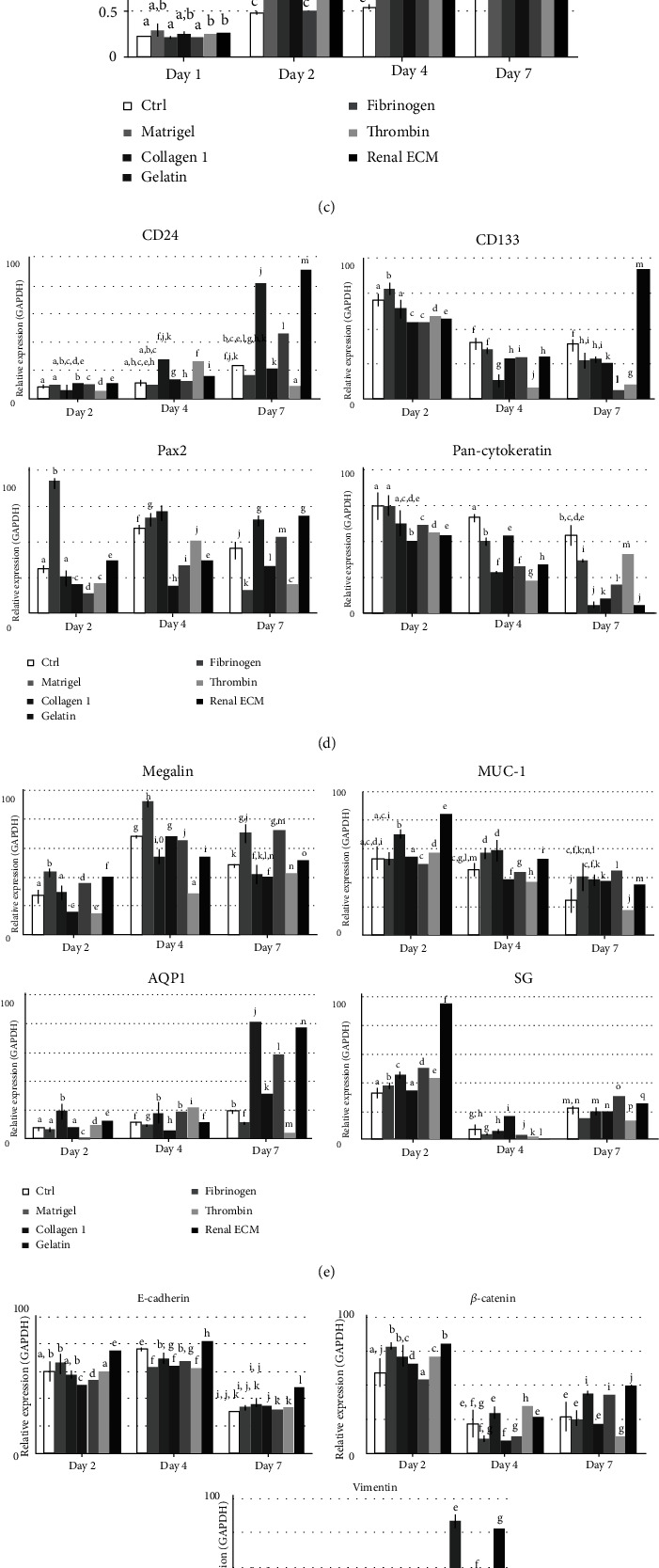
Effects of renal ECM as a coating material for human renal proximal tubular epithelial (RPTE) cell morphology, proliferation rate, gene expression, and senescence. Cells were cultured on the renal ECM-coated plates for 7 days. Coating materials, such as Matrigel, rat tail collagen type 1, gelatin, fibrinogen, and thrombin were compared. (a) Cell morphology on uncoated plate; (b) cell morphology on coated plate and cell assembly (arrowhead); (c) cell proliferation rate with MTT assay; (d–f) gene expression for human kidney-specific progenitors (CD133, CD24), representative renal progenitor (Pax2), general physiological features (pan-Cytokeratin), endocytosis receptor (Megalin), ion channels (Muc-1), water channel (AQP1), sodium-dependent glucose transport system (SGLT2), dedifferentiation from final differentiated epithelium to renal progenitor, and epithelial-to-mesenchymal transition (EMT) with E-cadherin, *β*-catenin, and Vimentin; (g) cell senescence assay. Renal ECM: porcine kidney-derived extracellular matrix; original magnification, 200; scale bar, 30 *μ*m. ←, swollen cell body; ▲, dendrite production; 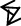, bipolar cell shape. Different letters above bars indicate significant differences (*p* < 0.05).

The effects as a coating material were compared with commercial coating materials. The representative images were taken at days 2, 4, and 7 with a bright-field microscope. The uncoated plate showed little proliferation for 7-day culture (Figure 1(a)). However, the plates coated with col1, thrombin, and renal ECM showed frequent cell proliferation, and the gathered cells form a cell assembly (arrowhead, Figure 1(b)). The cell assemblies varied in size and remained attached to the plate (did not float). The renal ECM and col1 group showed similar cell assembly morphology. The gelatin and fibrinogen groups formed relatively few and small cell assemblies. The cells cultured on fibrinogen-coated plates showed unclear cellular boundaries. The Matrigel-coated plates showed flat and stretched cell morphologies, and rapidly proliferated cells were elongated and produced a swirled pattern but did not form assembly for 7-day culture.

The MTT assay of cell proliferation (Figure 1(c)) showed that the RPTE cell proliferation was significantly increased in the renal ECM- and Matrigel-coated groups compared to others from day 2. At day 7, the Matrigel group showed the highest cell proliferation rate, to the next, col1, gelatin, and thrombin, and the renal ECM-coated group had similar proliferation rates. The fibrinogen-coated group had the lowest.

The renal-associated gene expressions were analyzed at each time point (Figures 1(d)–1(f)). CD24 and CD133 mRNA expression was significantly increased in the renal ECM-coated group at day 7. In the Pax2 gene analysis, a relatively higher expression was shown in the renal ECM and col1 group at day 7. The pan-Cytokeratin expression was lower in all the experimental groups than the uncoated group. Megalin was relatively highly expressed in the Matrigel and fibrinogen groups at day 7, and the renal ECM group was followed. Muc-1 expression was highest in the renal ECM group at day 2 and gradually decreased over time. AQP1 expression was significantly higher in the col1 and renal ECM groups at day 7. SGLT2 expression was the highest in the renal ECM group at day 2 and gradually decreased. E-cadherin and *β*-catenin expressions were highest in the renal ECM group at all times. Vimentin expression was the highest in the col1 and renal ECM group at day 7. Target gene expression was relatively high in the renal ECM group, and the col1 group showed similar expression patterns.

In the cell senescence assay (Figure 1(g)), the uncoated group showed positive blue stains at day 7 (arrow). The senescent cells showed a typically enlarged and flat morphology, reduced cytoplasmic volume, and contained debris. All experimental coating groups did not show cell senescence for 7-day culture.

### 3.2. Effective Renal ECM Concentration for Coating

**Figure 2:**
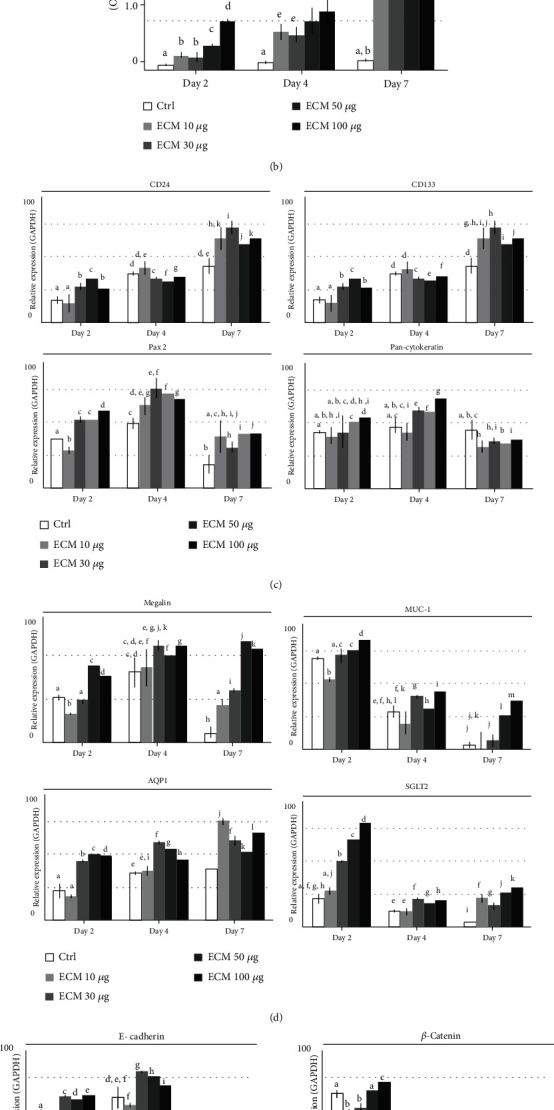
Effects of renal ECM concentration on human RPTE cell morphology, proliferation rate, and gene expression. Cells were cultured on the renal ECM-coated plate with various concentrations for 7 days. (a) Cell morphology, (b) cell proliferation rate, and (c–e) gene expression for CD133, CD24, Pax2, pan-Cytokeratin, Megalin, Muc-1, AQP1, SGLT2, E-cadherin, *β*-catenin, and Vimentin. ECM: porcine kidney-derived extracellular matrix; renal ECM concentration 10, 30, 50, or 100 *μ*g/*μ*L of each well of a 48-well plate. Original magnification, 200x; scale bar, 30 *μ*m; ←, swollen cell body; ▲, dendrite production; §, swirled pattern; ∗, mass formation. Different letters above bars indicate significant differences (*p* < 0.05).

After confirmation of renal ECM coating effects for cell proliferation and property maintenance, an effective concentration was then surveyed with cell proliferation rate. The surface of each well of a 48-well plate was coated with 10, 30, 50, or 100 *μ*g of renal ECM. Images were captured at days 2, 4, and 7 with a bright-field microscope (Figure 2(a)). When cells were counted manually, the uncoated plate's proliferation rate was 2.9% for 7-day culture. However, the renal ECM-coated plates showed a significantly increased cell proliferation depending on the ECM concentration. The ECM 10 *μ*g group showed 29.2% increase at day 2, and the ECM 100 *μ*g group could not be counted manually for cell assembly from day 2.

Morphologically, the uncoated plate (Figure 1(a)) showed ellipsoid (arrow), bipolarized (connected arrow), and dendrite production (arrowhead), and the round form cell numbers slowly increased by time course. The renal ECM-coated plate (Figure 2(a)) showed swollen cell bodies (arrow) and dendrite production (arrowhead) to connect to other cells on low cell densities. As the cell numbers increased, swirled patterns (§) appeared, and at high density, the cells showed mass formations (∗) by cell assembly, and the mass density increased over time. Depending on the ECM concentration, the cell assembly appeared early and dense.

In MTT analysis (Figure 2(b)), RPTE cell proliferation was significantly increased in all the renal ECM-coated groups compared to the uncoated group from day 2. The growth rate was dependent on the ECM concentration. At day 7, the proliferation gap between the coated and uncoated groups was further increased (*p* < 0.01).

The renal-associated gene expressions were then analyzed (Figures 2(c)–2(e)). The CD133 and CD24 mRNA expression was significantly increased in the renal ECM 30 *μ*g group at day 7, and the expression values were increased over time. In Pax2 gene analysis, a relatively high expression was shown at day 4 in the ECM 30 *μ*g group, and the expression had decreased at day 7. The pan-Cytokeratin gene was constantly expressed in the uncoated group, while renal ECM-coated groups showed an increased expression at day 4, and the highest expression was shown in the ECM 100 *μ*g group. In the analysis of epithelial functional property-related gene expression, Megalin, Muc-1, AQP1, and SGLT2 expressions were the highest in the ECM 50 *μ*g group at day 7, the ECM 100 *μ*g group at day 2, the ECM 10 *μ*g at day 7, and the ECM 100 *μ*g group at day 2, respectively. In the analysis of E-cadherin, *β*-catenin, and Vimentin, the expression was highest in the ECM 30 *μ*g group at day 4, the ECM 100 *μ*g group at day 2, and the ECM 100 *μ*g group at day 7, respectively.

Since the gene expressions were too diverse to select the optimal concentration, we decided the effective concentration range. The minimum effective concentration was 30 *μ*g, and the effect as a coating material was maintained to the maximum concentration (100 *μ*g/each well of the 48-well plate).

### 3.3. Cell Responses to the Renal ECM Hydrogel Material

**Figure 3:**
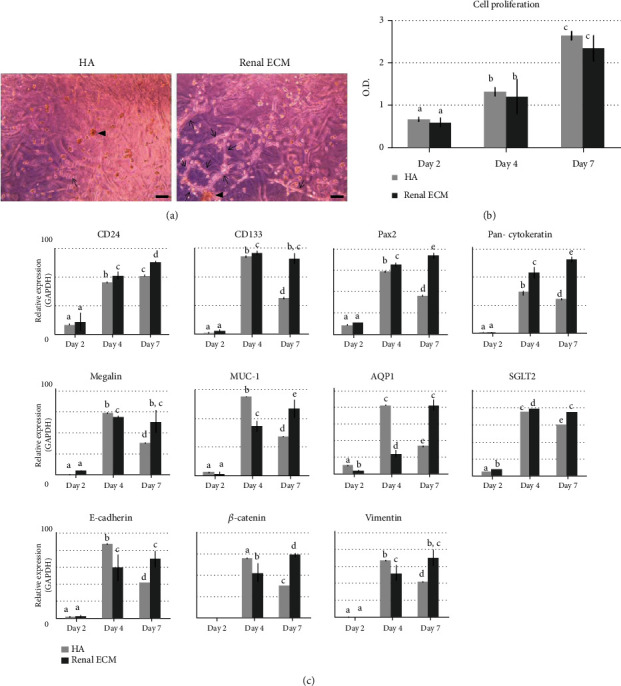
Effects of renal ECM as a hydrogel material on human RPTE cell morphology, proliferation rate, and gene expression. Cells were cultured in the renal ECM hydrogel (1 mg) for 7 days. Hyaluronic acid (1 mg) was used as a control. (a) Cell morphology in the hydrogel, (b) cell proliferation rate and (c) gene expression for CD133, CD24, Pax2, pan-Cytokeratin, Megalin, Muc-1, AQP1, SGLT2, E-cadherin, *β*-catenin, and Vimentin. ECM: porcine kidney-derived extracellular matrix; HA: hyaluronic acid. Original magnification, 100x; scale bar, 100 *μ*m. Different letters above bars indicate significant differences (*p* < 0.05).

The RPTE cell responses to renal ECM hydrogel were analyzed based on morphology, proliferation, and renal-related gene expressions, and each value was compared with the HA hydrogel. In morphological appearance at day 7 (Figure 3(a)), the renal ECM hydrogel showed frequently formed inner cell 3D networks (arrow) and large spheroid formation (arrowhead). In proliferation analysis (Figure 3(b)), RPTE cell proliferation was relatively decreased in the renal ECM hydrogel for 7-day culture. In renal-related gene expressions, renal ECM hydrogel showed higher expressions for all genes than the HA hydrogel at day 7 (Figure 3(c)).

### 3.4. Cell Responses to the Renal ECM Scaffold Material

**Figure 4:**
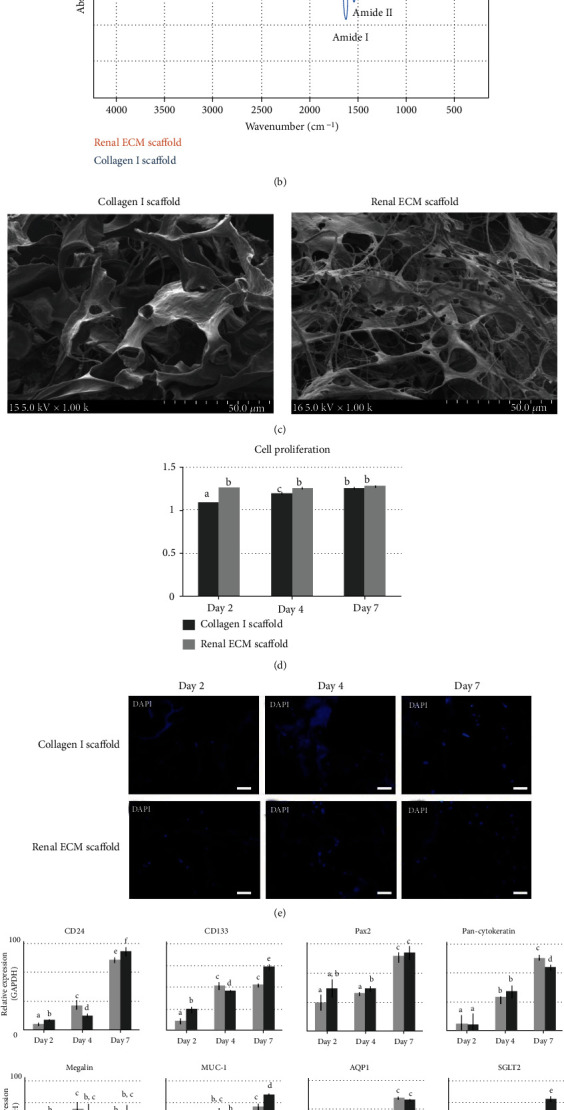
Effects of renal ECM as a scaffold material for human RPTE cell morphology, chemical composition, microstructure, proliferation rate, and gene expression. Cells were cultured on the scaffold (renal ECM concentration, 1 mg/scaffold) for 7 days. The collagen scaffold made from rat tail collagen type I scaffold was used as a control. (a) Scaffold morphology, (b) chemical composition analysis with FTIR spectroscopy, (c) SEM images, (d) cell proliferation rate, (e) cell distribution analysis with DAPI, and (f) gene expression for CD133, CD24, Pax2, pan-Cytokeratin, Megalin, Muc-1, AQP1, SGLT2, E-cadherin, *β*-catenin, and Vimentin. ECM: porcine kidney-derived extracellular matrix; collagen 1: scaffold made with rat tail collagen type I; renal ECM: scaffold made with renal ECM. Original magnification, 1000x; scale bar, 50 *μ*m. Different letters above bars indicate significant differences (*p* < 0.05).

The RPTE cell responses to renal ECM as a scaffold were compared to the rat tail type 1 collagen scaffold. The prepared renal ECM scaffold morphology was a whitened sponge form similar to the collagen scaffold (Figure 4(a)). The scaffolds were examined for chemical functional groups on the surface with FTIR (Figure 4(b)). The infrared bands of peptide linkages were found at the same location on both scaffolds. the peak for amide A (NH stretching) at 3300 cm^−1^, the peak for amide B (NH stretching) at 3100 cm^−1^, the peak for amide I (C=O stretching) at 1750 cm^−1^, the peak for amide II (CN stretching, NH bending) at 1535 cm^−1^, and the peak for amide III (CN stretching, NH bending) at 1230 cm^−1^. To evaluate the microstructure of the scaffold, scanning electron microscopy was performed on cross sections (Figure 4(c)). The renal ECM scaffold showed a 3D porous network, and the micronetwork fiber was thinner than the collagen scaffold, which results in high porosity. The porosity of renal ECM and collagen scaffold was about 98% and 95%, respectively.

Analyzing the cell proliferation rate of the scaffolds (Figure 4(d)), the renal ECM scaffold showed an enhanced proliferation rate at day 2 (*p* = 0.001). The gap gradually reduced over time, and the values were similar at day 7. The cell distribution in both scaffolds showed a similar pattern in the fluorescent DAPI stain (Figure 4(e)). Comparing renal-related gene expressions on both scaffolds, most genes were expressed more highly in the renal ECM scaffold than the collagen1 scaffold, except pan-Cytokeratin (Figure 4(f)).

### 3.5. Comparison of Renal Gene Expression according to Renal ECM Phase

**Figure 5:**
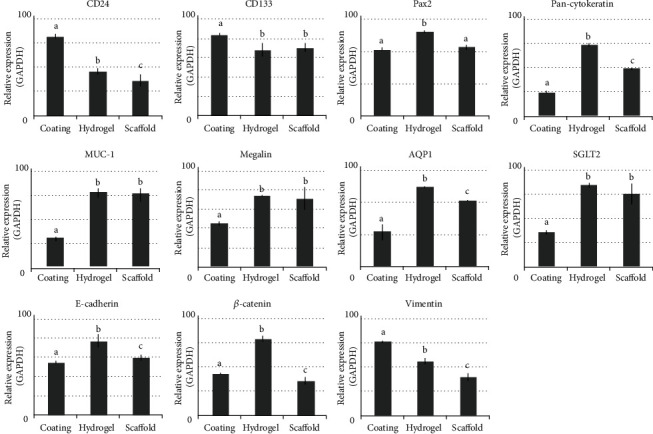
Comparison of renal-related gene expression according to renal ECM phase. Coating: coated phase of renal ECM; hydrogel: hydrogel phase of renal ECM; scaffold: freeze-dried scaffold phase of renal ECM. Different letters above bars indicate significant differences (*p* < 0.05).

Gene expressions according to the renal ECM phases were compared using PCR results at day 7 (Figure 5). The genes related to the renal progenitor state (CD24, CD133, and Vimentin) were highly expressed in the coating phase. Genes related to the renal epithelial function were significantly higher in the hydrogel and scaffold phase, and the expression was more prominent in the hydrogel.

## 4. Discussion

Although several methods for in vitro/ex vivo culture for RPTE cell have been suggested, the culture conditions still need improvement. In the medium composition, animal serum induces fibroblast overgrowth [13] and cytotoxic to mature RPTE cells [14]. It is hard to obtain the required cell numbers for in vivo therapy using a serum-free hormone medium with insulin, transferrin, and hydrocortisone [14]. The immortalized RPTE cell line with human papilloma virus (HPV 16) E6/E7 genes [15] cannot be used for clinical applications. Therefore, we devised new conditions using renal ECM as the coating, hydrogel, and scaffold phases. We expect that each renal ECM phase can provide the appropriate conditions for cell proliferation, property maintenance, 3D culture niche, and cell delivery vehicles.

First, renal ECM was applied as the coating material to enhance RPTE cell proliferation through enhanced adhesion. Surface adhesion is the important initiation process for in vitro/ex vivo cell culture. The anchorage-dependent RPTE cells could die if floating [16]. When attach to the surface, integrins on the cell membrane form an *αβ* heterodimer and combine with adhesion proteins on the surface. This combination generates cascade intracellular signals, and the genes for cell survival and proliferation are expressed [12]. In this process, adhesion proteins in the ECM are closely related to the cell anchorage. If adhesion molecules in the ECM are insufficient or inadequate for receptors, cells may float and undergo anoikic process, which is a programmed cell death process in anchorage-dependent cells [17]. The integrin subunits on the RPTE cell membrane are *α*1*β*1, which is a receptor for collagen; *α*3 is for collagen, laminin, and fibronectin; and *α*6 is for laminin. The RPTE cells also have separate receptors for elastin/laminin [18]. Considering that *α*-subunits specify ligand specificity, the renal ECM-coated surface composed of collagen, laminin, fibronectin, and elastin can promote RPTE cell binding through *α*-subunits. In the previous report [11], we confirm that these adhesion molecules were contained in the renal ECM abundantly.

As the coating material, the renal ECM effects were investigated with cell proliferation and expressed gene analysis. In MTT analyses, the RPTE cells cultured on the renal ECM coating surface showed the second-highest cell proliferation rate after Matrigel. During culture period, the renal ECM coating group showed two types of cell morphology, assembly and monolayer. The cell assembly may mean differentiation losing cell-cell contact inhibition [19]. However, according to Chung et al., it is a functional structure to solute transport by epithelial layer [14]). Thus, cell assemblies seen in the renal ECM group represent normal differentiation of RPTE cell. The cells remaining as monolayer were constantly proliferated, which could mean the stem cell character [19]. These two cell morphologies indicate a heterogeneous cell composition, and renal ECM coating can stimulate both renal progenitor proliferation and mature cell's full differentiation. Such morphological differences were related to the phenotypic result. In the renal ECM coating condition, the expressions of stem and renal progenitor cell markers (CD24, CD133, Pax2, and pan-Cytokeratin), mature differentiated tubular epithelial markers (Megalin, MUC-1, AQP1, and SGLT2), and dedifferentiation/epithelial-to-mesenchymal transition markers (E-cadherin, *β*-catenin, and Vimentin) were actively enhanced. These patterns of renal ECM coating for proliferation rate, cell morphology, and gene expressions were very similar to col1coating, which means that the renal ECM composing factors are mainly collagen. In the case of Matrigel, cell proliferation was high, but the functional characteristics as renal epithelial cells were significantly reduced. Gelatin and fibrin coating effects were not noticeable, contrary to the reported effect [19, 20]. Thus, we prove that renal ECM as a coating material is effective for RPTE cell proliferation maintaining cell functional properties compared to Matrigel, col1, gelatin, fibrinogen, and thrombin coating.

After proof of the benefit of renal ECM as a coating material, the optimal coating concentration was surveyed. The ECM concentration is one of the key factors to induce appropriate cellular responses, because ECM protein concentrations are closely related to biochemical and biophysical cell properties [21]. Each molecule constituting the ECM has different adhesion forces resulting in different cell migration speed; e.g., fibronectin exhibits a higher cell migration force than laminin [22]. Also, excessive ECM concentration increases rigidity and interstitial pressure, which causes dysregulation of ECM remodeling and homeostasis [23]. ECM concentration also influences biophysical contractile forces through the control of fibrous proteins (such as collagen, elastin, and laminin) [21], which affects diffusive transport for water, minerals, proteoglycans, and waste products.

To select the optimum ECM coating concentration, the culture dish surface was coated with 10, 30, 50, or 100 *μ*g of renal ECM per well of a 48-well plate. In morphological appearance, depending on the ECM concentration, cell proliferation was increased, a swirled pattern appeared early, and cell assembly appeared early and intensively. These morphological patterns were generally connected to the regulation of gene transcription. However, the gene expression patterns were very diverse according to gene type, ECM concentration, and culture period, so it was hard to determine the exact optimal concentration. Thus, the effective concentration was specified as a range; the minimum effective concentration started at 30 *μ*g and the maximum concentration was up to 100 *μ*g per well of a 48-well plate.

Second, renal ECM was further applied as a hydrogel phase to provide an in vivo-mimic niche to maintain RPTE cell functionality. In actual in vivo condition, the bottom of the RPTE cell is attached to the basement membrane composing ECM. Both sides of the cell face neighboring cells via tight junctions, adhering junctions, and desmosomes [24]. The apical side of the cell faces the lumen and polarized with a brush border of microvilli [25]. As we know, the 2D culture cannot form a cuboidal cell shape, and it is hard for the flat cells to receive the signals through membrane receptors [26], which results in abnormal polarization and loss of phenotypic potential [27], while the spatial organized cell's receptors interact with neighboring cells regulating the gene expressions and cellular functions for reabsorption and secretion of ions and macromolecules [28]. To maintain the RPTE cell functionality, the 3D hydrogel phase is preferable. The hydrogel state has several advantages, e.g., increased biocompatibility, tunable biodegradability, mechanical strength, porous structure for small molecule penetration, stable covalent bond formation within proteins, promotion of cell migration, and 3D tissue construction [29]. Thus, we manufactured the hydrogel with renal ECM. The renal ECM hydrogel is a self-assembled gel formed by physical crosslinking without any toxic chemical process, and the main component is a collagen [11]. We expect that the collagen-based renal ECM hydrogel can present renal-specific ligands resulting in high renal cell adhesivity and similar structural/mechanical properties as the native kidney.

To analyze the hydrogel effect of renal ECM, HA hydrogel was used as the control [27]. In morphological observation, renal ECM hydrogel showed a well-composed tubule-like 3D structure, while the HA gel showed a less formed structure, which enables the renal ECM hydrogel to provide a kidney proximal tubule-mimic basement and stiffness resulting in appropriate cell migration. In the renal ECM hydrogel, the embedded RPTE cell proliferation rate was relatively lower than HA. However, in renal-associated gene expressions, the cultured RPTE cell in the renal ECM hydrogel significantly increased in all gene's expression at day 7. These results indicate that the renal ECM hydrogel is very useful material to maintain renal epithelial characteristics; thus, this hydrogel phase can be applied for 3D cell culture and delivery device.

Third, renal ECM was further applied as the scaffold material, because dissected tissue augmentation and structural strength cannot satisfy with hydrogel filling. Also, the scaffold phase can maintain renal tubular structures and more effective for monolayer formation of basolateral and apical cubicles than hydrogel phase [30]. Other benefits are long-term storage, room temperature handling, and convenience of transport regardless of temperature. Further, the scaffold can be molded to the desired shape, has a simple sterilization process, provides long-term maintenance of protein and growth factor activity and various fabrications, and allows for diverse surface modification [31].

The renal ECM scaffold was prepared by a simple freeze-drying process. The final product showed a white sponge-like morphology that is similar to the col1 scaffold. In FTIR analysis, the renal ECM scaffold showed specific amide A, B, I, II, and III peaks, which means that the renal ECM has functional groups for surface modification. In SEM images, the renal ECM scaffold showed a well-organized microstructure with high porosity, which means that the medium components (nutrients) can easily diffuse into the scaffold, and cell waste products are quickly removed from the scaffold. Also, the expanded surface by the thin fiber favors cell adhesion. In the analysis of the cell proliferation rate, the renal ECM scaffold showed a similar value to the col1 scaffold. In cell distribution analysis with DAPI, the renal ECM scaffold showed a relatively frequent DAPI positive distribution. The small bright dots with DAPI stain indicate nucleus in a cell, and the broad faint blue color is the spontaneous fluorescence of formalin-fixed biological material with wavelengths similar to DAPI. In real-time PCR analysis, most of the renal-related genes were highly expressed in the renal ECM scaffold. These results mean that the renal ECM scaffold can also provide a suitable microenvironment for RPTE cells' 3D cell culture and delivery device.

Finally, we compared the gene expressions of the RPTE cell in each renal ECM phase, because cells constantly interact with the ECM and synthesize different active molecules according to ECM phases, which directly affects the cell fate and its functions [32]. So far, there has been no report comparing the RPTE cellular response in three different phases of renal ECM. In our study, the genes related to the renal progenitor state (CD24, CD133, and Vimentin) were highly expressed in the coating phase. Gene expression for physiological cell polarization and microvilli formation was more enhanced in the hydrogel and scaffold phase; the value was more prominent in the hydrogel phase. Therefore, these results indicate that the coating phase of renal ECM is preferable for proliferation of RPTE cells, and the hydrogel and scaffold phases are suitable for functional property maintenance, when used as 3D culture niche and cell delivery vehicles. We hope that these renal ECM phases will provide a roadmap for research using RPTE cells.

For future studies, manufacture of bioartificial kidney device with electrospun, nano-/microparticles, spheroids, organoids, and encapsulation with renal ECM are ongoing. Also, for preclinical application, renal ECM hydrogel and scaffold seeding RPTE cell are directly administrated to the injured animal kidney. Further, physical analysis, e.g., mechanics, swelling, mesh size, and degradation, is going to be analyzed.

## 5. Conclusion

Renal ECM has a suitable composition for RPTE cell proliferation and property maintenance providing an in vivo-mimic niche. The renal ECM effects were superior to those of col1, Matrigel, gelatin, fibrinogen, thrombin, and HA. As a practical guide, the coating phase is suitable for RPTE cell enhanced proliferation. The hydrogel and scaffold phases can provide RPTE cell's functional property maintenance, when applied as 3D culture material and cell delivery vehicle.

## Figures and Tables

**Table 1:** The primer sequences.

Gene	Function	Sequences
CD24	Human kidney-specific progenitors	5′-tca aca gcc agt ctc tc gt-3′ 5′-gac gtt tct tgg cct gag tc-3′
CD133		5′-ttc ttg acc gac tga gac cc-3′ 5′-tgg tct cct tga tcg ctg tt-3′

Paired box gene 2 (Pax2)	Representative renal progenitor	5′-tct ctc ctc tcc gct tct ct-3′ 5′-cga cag aga cgg aga ac-3′

Pan-Cytokeratin	General physiological feature	5′-act tga caa ctt gca gca gg-3′ 5-caa tga tgc tgt cca ggt cg-3′

Megalin	Endocytosis receptor	5′-ctt gca act atc cga cct gc-3′ 5′-gga ccg ctt tca cat cca tc-3′

Mucin-1 (Muc-1)	Ion channels	5′-tcc ttt ctc tgc cca gtc tg-3′ 5′-caa cca gaa cac aga cca gc-3′

Aquaporin 1 (AQP1)	Water channel	5′-cca tca caa ctc tcc cca ct-3′ 5′-cca tca caa ctc tcc cca ct-3′

Sodium glucose co-transporter 2 (SGLT2)	Sodium-dependent glucose transport system	5′-ctc tct tcg cca gca aca tc-3′ 5′-cca ctc gaa tcc agc aac ag-3′

Epithelial cadherin (e-cadherin)	Epithelial-to-mesenchymal transition markers	5′-aag ggg tct gtc atg gaa gg-3′ 5′-ggtgtt cac atc atc gtc cg-3′
*β*-Catenin		5′-gag ggt acg agc tgc tat gt-3′ 5′-aac gct gga cat tag tgg ga-3′
Vimentin		5′-ctt tgc cgt tga agc tgc ta-3′ 5′-acg agc cat ttc ctc ctt ca-3′

## Data Availability

Data is available on request.
